# The Association of Fasting Glucose and Total Cholesterol with Cognitive Performance in a Primary Care Population: A Cross-Sectional Study

**DOI:** 10.3390/jcm15197391

**Published:** 2026-09-23

**Authors:** Agnieszka Gostyńska, Anna Posadzy-Małaczyńska, Nadia Kruszyńska, Małgorzata Romanowska, Agata Puszcz, Karolina Piesiak, Tomasz Konopelski, Barbara Ostrowska

**Affiliations:** 1 Faculty of Finance and Banking, Merito University of Poznań, 5 Powstańców Wielkopolskich Street, 61-895 Poznan, Poland; 2 Department of Family Medicine, Poznań University of Medical Sciences, 70 Bukowska Street, 60-812 Poznan, Poland; 3 Sexology and Clinical Psychology Student Research Group, Department of Clinical Psychology, Poznań University of Medical Sciences, 70 Bukowska Street, 60-812 Poznan, Poland; 86535@student.ump.edu.pl

**Keywords:** neurocognitive disorders, mild cognitive impairment, dementia, primary care

## Abstract

**Objectives:** This cross-sectional study aimed to evaluate the associations between fundamental metabolic markers, specifically fasting glucose and total cholesterol, and cognitive performance in a primary care population, continuing previous analyses focused on subclinical vascular parameters. **Methods:** The study included 137 primary care patients who completed neuropsychological assessments (Addenbrooke’s Cognitive Examination-III (ACE-III) and Mini-Mental State Examination (MMSE)) and a clinical interview. Fasting glucose and total cholesterol levels were extracted from their medical records. **Results:** Based on the MMSE, 26 participants (19.0%) had mild cognitive impairment (MCI), and 8 (5.8%) had neurocognitive disorders. Univariate regression analysis revealed that fasting glucose was significantly and inversely associated with the total ACE-III score (B = 0.277; 95% CI: −0.441 to −0.113; *p* = 0.001) and the M-ACE subscale score (B = 0.175; 95% CI: −0.342 to −0.008; *p* = 0.046). Conversely, total cholesterol showed a positive association with both the overall ACE-III score (B = 0.173; 95% CI: 0.006 to 0.340; *p* = 0.046) and the M-ACE score (B = 0.219; 95% CI: 0.053 to 0.385; *p* = 0.011). A potential clinical paradox was observed, as among patients with established cardiovascular disease, lower total cholesterol (177.39 vs. 201.58 mg/dL, *p* = 0.047) co-occurred with poorer cognitive outcomes (mean ACE-III: 81.47 vs. 87.62, *p* = 0.003), possibly reflecting a confounding effect of statin therapy. Standard MMSE scores did not correlate significantly with any evaluated metabolic parameters. **Conclusions**: Fasting glucose and total cholesterol are cross-sectionally associated with cognitive performance in this primary care cohort, whereas standard MMSE scores did not exhibit significant metabolic correlations. Given the exploratory, cross-sectional design, these findings are strictly hypothesis-generating and do not establish clinical screening utility or causality. The disparate patterns of association between the ACE-III and MMSE suggest that these screening tools may capture metabolism-related cognitive variations differently, highlighting the need for prospective longitudinal research.

## 1. Introduction

The escalating prevalence of dementia syndromes represents a formidable challenge in primary care practice. As the global population ages, the epidemiological burden of Alzheimer’s disease and other dementias (ADOD) has surged dramatically, with a particularly pronounced increase observed in individuals aged 60 to 74 years. This expanding global health issue imposes a substantial financial and societal burden [1]. In response to this public health crisis, recent global initiatives, such as the updated 2026 World Health Organization (WHO) guidelines on risk reduction, emphasize that addressing modifiable risk factors across the lifespan remains the most effective strategy to reduce future dementia incidence [2]. However, existing studies indicate that primary care physicians frequently omit routine cognitive screening in older adults, leading to severely delayed diagnoses [3]. Mild cognitive impairment (MCI) constitutes an intermediate, heterogeneous clinical state between normal cognitive aging and early dementia. Patients typically present with subjective memory complaints, such as misplacing objects or forgetting appointments, that do not yet compromise their independence in daily activities. Nevertheless, without intervention, individuals with MCI face a substantially heightened risk of progressing to severe neurocognitive disorders [4]. Objective standardized assessments, such as the Mini-Mental State Examination (MMSE) and Addenbrooke’s Cognitive Examination-III (ACE-III), are essential for detecting these subtle deficits in clinical settings. In primary care, detailed cognitive evaluations are frequently constrained by limited time and clinical resources. Consequently, the identification of easily accessible, routine biomarkers is critical for early risk stratification [3]. The 2010 American Heart Association’s Life’s Simple 7 criteria emphasize that optimizing fundamental cardiovascular and metabolic parameters, such as fasting blood glucose and cholesterol, plays a vital protective role against both cardiovascular events and neurodegenerative processes [5]. Recent evidence underscores that glycemic control is crucial for preserving cognitive health [6]. High fasting plasma glucose (HFPG) has recently been identified as a leading, modifiable risk factor for ADOD [1]. Chronic hyperglycemia, even at prediabetic levels, correlates with a faster rate of global cognitive decline and microstructural brain damage [7,8]. Furthermore, systematic reviews demonstrate that acute glycemic variability significantly increases the risk of cognitive impairment [9]. Abrupt glucose fluctuations induce vascular damage and neuroinflammation, ultimately resulting in neuronal injury [10]. Conversely, strict glycemic control through targeted interventions has been shown to significantly attenuate the progression of cognitive decline [6].

Alongside glucose homeostasis, lipid profiles represent another crucial, yet complex, metabolic window into brain health. While midlife dyslipidemia is an established risk factor for future cognitive impairment, recent longitudinal data highlight the importance of intraindividual lipid fluctuations in older adults. High year-to-year variability in total cholesterol (TC) and low-density lipoprotein (LDL), independent of mean lipid levels or lipid-lowering medication changes, has emerged as a robust predictor of accelerated cognitive decline and incident dementia. These late-life fluctuations may reflect reduced physiologic reserve, systemic comorbidities, or functional decline, leading to compromised cerebral perfusion and microvascular damage. Early detection of metabolic dysregulations offers a critical window for implementing preventive lifestyle and pharmacological strategies to preserve neurocognitive function [4,11].

The present study constitutes a continuation of previous analyses based on the same research project, which evaluated the relationship between the ankle–brachial index (ABI) and cognitive function. Whereas the previous study focused on subclinical vascular parameters, the current analysis aims to evaluate the associations between fundamental metabolic markers, specifically glucose and cholesterol concentrations, and cognitive performance in the examined population [12].

We formulated the following research hypotheses:

(1) Higher total cholesterol (TC) concentrations are significantly and positively associated with cognitive scores in this cross-sectional older adult cohort, although the relationship in older adults may be complex and influenced by the presence of cardiovascular disease and its treatment [4].
(2) Elevated fasting blood glucose (FBG) levels are inversely associated with global cognitive performance in a primary care population, even among individuals without a formal diagnosis of diabetes [13].
(3) The comprehensive ACE-III assessment may capture subtle metabolism-related cognitive variations differently from the standard MMSE screening, reflecting potentially disparate descriptive capacities in this setting [3,14].

The rationale for selecting total cholesterol as the primary lipid marker in this analysis is based on its role as a fundamental, routinely available parameter in primary care that provides a critical early window into systemic metabolic health. Furthermore, TC is a core component of established cardiovascular health criteria, such as the AHA Life’s Simple 7 [5], and its late-life fluctuations have emerged as a robust indicator of physiological reserve and neurocognitive risk, making it a highly practical tool for initial risk stratification in general practice [11].

## 2. Materials and Methods

The present study represents a continuation of broader research [12] conducted by Agnieszka Gostyńska et al. as part of her doctoral dissertation preparation and is based on the cohort analyzed in a previous publication examining the association between the ankle–brachial index (ABI) and cognitive functioning. The current analysis extends the investigation by focusing on metabolic parameters, specifically fasting glucose and total cholesterol concentrations, and their relationship with cognitive performance. This cross-sectional observational study was conducted in a primary care setting. A total of 137 participants were consecutively recruited from Family Medicine outpatient clinics at the Department of Family Medicine, Poznań University of Medical Sciences, who volunteered and provided written informed consent to participate in this study. Ethical approval was obtained from the Bioethics Committee of the Poznań University of Medical Sciences (resolution number 1061/16). Due to the exploratory nature of the project, no formal sample size estimation was performed. However, a retrospective post hoc power analysis indicated that our final sample size of 137 participants provided approximately 90% statistical power at an α significance level of 0.05 to detect the primary inverse association between fasting blood glucose and total ACE-III scores (R^2^ = 0.069).

Participants aged 50–76 years were specifically recruited to capture individuals at elevated risk for cognitive impairment. Recruitment and data collection were conducted between January and December 2017. Exclusion criteria comprised a history of unstable coronary artery disease, heart failure with left ventricular ejection fraction <40%, cardiomyopathy, cardiac arrhythmias (except in patients with an implanted pacemaker), structural heart defects, secondary hypertension, grade 3 hypertension (systolic blood pressure [SBP] ≥ 180 mmHg and/or diastolic blood pressure [DBP] ≥ 110 mmHg, according to European Society of Hypertension [ESH] guidelines), malignant neoplasms, hematological disorders, hepatic cirrhosis, renal failure, neurological diseases, substance dependence (including alcohol, drugs, or medication), previously diagnosed cognitive impairment, depression or other psychiatric conditions with currently exacerbated symptoms, and visual or auditory impairments that precluded the completion of cognitive assessments.

To ensure a representative yet cognitively testable primary care cohort, the exclusion criterion of ‘neurological diseases’ specifically referred to acute, unstable, or progressive central nervous system (CNS) disorders (e.g., primary CNS malignancies, recent stroke, or advanced demyelinating diseases) as well as previously documented clinical dementia. Conversely, stable, chronic, and well-controlled neurological conditions that did not interfere with the participants’ capacity to undergo cognitive testing (such as long-term controlled epilepsy or chronic stable headaches) were permitted, accounting for the 8 participants (5.84%) with a history of stable CNS conditions reported in the baseline characteristics.

A flowchart illustrating the number of participants assessed, excluded, and included in the final analysis is presented in Figure 1.

The final analysis included 137 participants, as 8 of the 145 individuals who completed the study protocol were subsequently excluded due to the post-enrollment identification of missing contemporaneous fasting blood glucose or total cholesterol measurements within the strict 6-month window prior to the neurocognitive assessment. To ensure that excluding the 8 participants did not introduce selection bias, their baseline characteristics were compared with those of the analyzed cohort (*n* = 137). The excluded individuals exhibited comparable mean age (64.1 ± 5.8 vs. 63.5 ± 5.5 years), sex distribution (50.0% vs. 53.3% female), and MMSE scores (27.1 ± 2.1 vs. 27.2 ± 2.3), confirming the absence of evident selection bias.

The study procedure consisted of three consecutive stages. During the first stage, participants underwent an initial interview and provided written consent. Demographic characteristics, socioeconomic information, previous medical history, and risk factors were collected. Medical records were additionally reviewed to extract anthropometric measurements, laboratory parameters, and relevant clinical information.

The second stage included cognitive assessment using the Mini-Mental State Examination (MMSE) and Addenbrooke’s Cognitive Examination III (ACE-III) [14]. All assessments were administered in a quiet, dedicated consultation room by a single researcher formally trained in neuropsychological test administration. To ensure cross-cultural validity, officially validated Polish versions of both instruments were employed: the MMSE (published by the Psychological Test Laboratory of the Polish Psychological Association, 2010 [15]) and the ACE-III (adapted by Sitek et al. [16]). The standardized testing sequence was identical for all participants, commencing with the structured clinical interview, followed by the MMSE, and concluding with the ACE-III.

Cognitive status was classified using established MMSE cut-offs. Based on previous literature, including validation by Tsai et al. [17], scores of 24–26 were indicative of mild cognitive impairment (MCI). For probable severe neurocognitive disorders (NCDs) reaching the dementia level, a threshold of ≤23 was applied in accordance with Chapman et al. [18], which aligns with criteria demonstrating a high positive predictive value for Alzheimer’s disease. Raw, unadjusted cognitive scores were used for classification. Although participants’ age and formal education level were analyzed as descriptive cohort characteristics and evaluated in baseline group-difference analyses, the primary linear regression models were conducted as unadjusted (univariate) exploratory analyses.

Additionally, the Mini-Addenbrooke’s Cognitive Examination (M-ACE) [14] was incorporated as an exploratory secondary endpoint. Importantly, the M-ACE was not administered as an independent test; instead, its 30-point score was derived directly from the corresponding items of the completed 100-point ACE-III questionnaire, following validated guidelines [14]. Only participants who completed all items of the cognitive protocols with no missing responses were included in the final analysis (complete-case approach).

The third stage involved vascular assessment using a BOSO ABI-System 100 device (BOSO MedTech, Jungingen, Germany), which has been validated for non-invasive cardiovascular measurements. Parameters recorded included the ankle–brachial index (ABI), pulse wave velocity (PWV), and blood pressure measurements.

Metabolic parameters, including fasting blood glucose (FBG) and total cholesterol (TC), were extracted from standardized electronic medical records. To ensure data quality and comparability, only laboratory findings were extracted within a maximum interval of 6 months immediately preceding the neurocognitive assessment. For each participant, FBG and TC values were selected from the same routine morning venous blood draw, documented in the medical records as having been performed after an overnight fast of at least 8 h [3]. This protocol strictly adhered to the clinical guidelines of Diabetes Poland (PTD) [19]. Baseline metabolic profiles were contextualized against national clinical standards: impaired fasting glucose and diabetes were categorized using cut-offs from the 2026 Diabetes Poland (PTD) guidelines (normal fasting glucose < 100 mg/dL [5.56 mmol/L], impaired fasting glucose 100–125 mg/dL [5.56–6.94 mmol/L], diabetes ≥ 126 mg/dL [≥7.0 mmol/L]) [19], while dyslipidemia was referenced against the 2021 Polish Dyslipidemia Guidelines (desirable total cholesterol < 190 mg/dL [<4.9 mmol/L]) [19]. All biochemical analyses were processed in the same certified central laboratory of the university hospital using standardized, automated enzymatic assays to ensure full technical and methodological comparability across all measurements. Complete-case analysis was strictly applied, and any participant lacking verified FBG or TC values within the specified 6-month window was excluded during the screening phase, resulting in no missing values for these primary metabolic exposures in the final analyzed cohort (*n* = 137) [3].

The variables analyzed in this study were categorized into dependent and independent parameters to ensure methodological and statistical consistency.

The dependent variables (outcomes) consisted of the cognitive assessment scores, specifically the ACE-III total score, M-ACE subscale, and MMSE score.

The primary independent variables (predictors of interest) were metabolic parameters extracted from the patients’ medical records, specifically focusing on fasting glucose concentrations and total cholesterol levels. To account for potential confounding, interview-derived data concerning shared risk factors for cardiovascular disease (CVD) and neurocognitive disorders (NCDs) were incorporated into the exploratory models as additional independent predictors and covariates. These data encompassed current or past chronic conditions, prior CVD episodes, familial predisposition, lifestyle factors (tobacco and alcohol consumption), socioeconomic status, and current pharmacotherapy [12].

Sociodemographic characteristics were incorporated as covariates in the analysis. These encompassed the participants’ age, sex, formal education level (including years of schooling and high school diploma attainment), occupational history (manual versus intellectual labor), and current employment status. All statistical analyses were conducted using IBM SPSS Statistics (version 24.0; IBM Corp., Armonk, NY, USA). Continuous and categorical data were summarized using standard descriptive statistics, including means, standard deviations, frequencies, percentages, and minimum/maximum values. Due to the non-normal distribution of continuous variables, the non-parametric Mann–Whitney U test was employed for group comparisons, whereas Dunnett’s T3 test was utilized for post hoc variance analysis. To evaluate the relationships between metabolic parameters and cognitive performance, Pearson’s correlation coefficient (r) was calculated, followed by univariate linear regression analysis to evaluate unadjusted associations. No adjustments for multiplicity were applied across statistical comparisons; thus, all findings remain strictly exploratory. Given the observational cross-sectional design, no causal inferences can be drawn regarding the protective or harmful effects of these metabolic parameters.

## 3. Results

The participants’ psychosocial and socioeconomic profiles were evaluated using a structured questionnaire. This assessment focused on established risk factors for cognitive impairment (such as mood disturbances, psychological trauma, recent bereavement, social isolation, and lifestyle habits) and is summarized in Table 1 and Table 2. In our analysis, regular computer use was classified as a protective factor, given its potential contribution to the maintenance of cognitive function.

Global cognitive functioning was evaluated using standardized psychological tests. The mean performance on the MMSE reached 28.07 (SD = 5.11) points. According to established MMSE cut-off values, scores greater than 26 reflect normal cognitive function, a range of 24–26 signifies MCI, whereas results below 24 suggest probable NCD. Based on these criteria, the study revealed that 75.18% of participants (*n* = 103) exhibited no cognitive deficits. MCI was detected in 18.98% (*n* = 26) of the study sample, and 5.84% (*n* = 8) presented scores indicative of NCD. Furthermore, the comprehensive ACE-III assessment resulted in an average overall score of 86.04 (SD = 11.04), with the M-ACE subscale yielding a mean of 24.20 (SD = 4.79).

To evaluate the relationship between metabolic parameters and cognitive performance, the Pearson correlation coefficient (r) was calculated. A statistically significant negative correlation was observed between fasting glucose levels and the total ACE-III score (r = −0.28, *p* < 0.01). This finding indicates that higher fasting glucose concentrations are associated with poorer global cognitive functioning. Additionally, a weak but significant positive correlation was found between total cholesterol levels and the ACE-III score (r = 0.17, *p* < 0.05), suggesting that higher cholesterol was linked to slightly better cognitive performance in this specific cohort. In contrast, no significant correlations were observed between standard MMSE scores and any of the evaluated metabolic parameters, including fasting blood glucose. To formally test whether the association patterns differed significantly between cognitive instruments, Steiger’s Z-test for comparing dependent correlation coefficients was applied. The correlation between fasting glucose and the ACE-III (r = −0.277) was significantly stronger than that between fasting glucose and MMSE (r = −0.082; Z = −2.14, *p* = 0.032), statistically confirming a disparate sensitivity pattern between the comprehensive ACE-III and the standard MMSE screening tool.

Subsequent univariate regression analyses were performed to evaluate the simple linear associations between these metabolic markers and cognitive outcomes. The analysis revealed that fasting glucose exhibited a weak but significant negative association with the total ACE-III score (β = −0.28, 95% CI: −0.44 to −0.11, *p* = 0.001; adjusted R^2^ = 0.069) and the M-ACE subscale score (β = −0.18, 95% CI: −0.35 to −0.01, *p* < 0.05; adjusted R^2^ = 0.022) (Figure 2a and Figure 3a). Conversely, total cholesterol showed a weak but positive association with both the total ACE-III score (β = 0.17, 95% CI: 0.01 to 0.34, *p* < 0.05; adjusted R^2^ = 0.022) and the M-ACE score (β = 0.22, 95% CI: 0.05 to 0.39, *p* = 0.011; adjusted R^2^ = 0.041) (Figure 2b and Figure 3b). This positive association between cholesterol and cognitive performance in this specific cohort should be interpreted cautiously, as the presented data do not establish that statin therapy explains the observed relationship. To visualize the relationship between metabolic parameters (expressed in mg/dL) and total ACE-III scores, regression lines with 95% confidence bands and reported β coefficients were plotted (Figure 2 and Figure 3).

Furthermore, as detailed in our previously published analysis of this cohort [12], specific lifestyle and vascular factors were also significantly associated with cognitive outcomes: healthy lifestyle habits (such as regular physical activity and computer use) and better psychosocial well-being were associated with higher MMSE and ACE-III scores, whereas subclinical vascular damage—reflected by lower ABI values—correlated with poorer cognitive performance.

## 4. Discussion

The purpose of this study was to investigate the association between global cognitive impairment and underlying metabolic disturbances evaluated by fasting glucose and total cholesterol concentrations. These biochemical markers are commonly used in primary care as fundamental, non-invasive indicators of overall cardiometabolic and metabolic health. Fasting blood glucose (FBG) represents the concentration of circulating glucose, determined via standard venous blood sampling, following an overnight fast. According to the 2026 Polish Diabetes Association guidelines [19], physiologically appropriate FBG levels are established strictly below 100 mg/dL (70–99 mg/dL). Although these specific reference ranges may vary slightly depending on regional or international guidelines [20], the average FBG concentration observed in the study group significantly exceeded these thresholds. Total cholesterol (TC) represents the sum of cholesterol contained within high-density lipoprotein (HDL), low-density lipoprotein (LDL), and very-low-density lipoprotein (VLDL). It is quantified via standard venous blood analysis. According to the 2021 Polish guidelines for the management of dyslipidemia, optimal TC levels are recommended to remain strictly below 190 mg/dL [21]. These specific parameters were selected for this study because their routine evaluation in primary care provides a critical early window into the systemic cardiometabolic dysregulations that subtly but profoundly impair cognitive functions. The potential clinical value of routine metabolic markers for preliminary cognitive risk stratification in primary care remains strictly at the hypothesis-generating stage. Rigorous prospective longitudinal validation with repeated metabolic and cognitive tracking is required before these routine parameters can serve as formal clinical screening indicators.

The association between FBG levels and cognitive disorders remains a critical area requiring further exploration. In a large cross-sectional study published in 2020, Liu et al. examined 807 middle- and advanced-age Japanese male participants, 191 diabetic and 616 without diabetes. The researchers used the Cognitive Ability Screening Instrument (CASI) to assess participants’ cognitive functions. A significant correlation between the level of FBG and CASI score was found in both diabetic and non-diabetic participants. In diabetic individuals, each 1 mmol/L FBG increase was associated with a significant decline in cognitive performance (a 0.38-point decrease in CASI score), with short-term memory being the most adversely affected domain. Conversely, in non-diabetic individuals, this relationship followed an inverted U-shaped curve, revealing an optimal glycemic range (71.5–111.6 mg/dL) for peak cognitive performance, beyond which cognitive function, notably language functions, deteriorated [22]. To establish a temporal relationship beyond cross-sectional observations, Shimoda et al. conducted a prospective cohort study between 2002 and 2018, investigating the impact of midlife FBG levels on cognitive function in later years. The researchers used the Montreal Cognitive Assessment (MoCA) to assess the cognitive performance of 253 participants in the study. Elevated midlife FBG levels were found to be an independent, negative predictor of future cognitive performance, even after adjusting for multiple confounders such as blood pressure, BMI, and cholesterol levels. Specifically, the multivariable-adjusted mean MoCA scores progressively declined from 25.2 in normoglycemic individuals to 24.8 in those with impaired fasting glucose and dropped significantly to 23.4 in patients with diabetes. Of particular concern, individuals with diabetic FBG levels had a more than three-fold increased risk (OR = 3.29) of developing mild cognitive impairment (defined as a MoCA score ≤ 25) after a 16-year follow-up, a deficit estimated by the authors to be equivalent to approximately 8 years of cognitive aging [13].

The findings from the present study align with these global observations. Consistent with established literature, our analysis demonstrated a significant inverse correlation between FBG levels and overall cognitive performance. Specifically, the univariate linear regression analysis revealed a significant negative association between fasting glucose levels and the total ACE-III score (Figure 2a). In contrast, no such relationship was observed when using the Mini-Mental State Examination (MMSE). While this descriptive discrepancy might lead to the hypothesis that a more comprehensive instrument such as the ACE-III is better suited to capture subtle metabolism-related cognitive variations in a primary care setting than the brief MMSE, this observation must be interpreted with caution. This observed difference in sensitivity was statistically confirmed by Steiger’s Z-test (Z = −2.14, *p* = 0.032), supporting the notion that the ACE-III captures subtle metabolism-related cognitive variations better than the MMSE in this setting. However, as no diagnostic ROC curves or sensitivity and specificity evaluations against a formal diagnostic standard were performed, claims regarding clinical diagnostic utility remain strictly exploratory and hypothesis-generating. Furthermore, these results must be interpreted with caution because the minimum fasting glucose in the study was 72 mg/dL, precluding the evaluation of hypoglycemia; as a result, our data only reflect associations across the euglycemic and hyperglycemic ranges.

Although the adverse effects of elevated fasting glucose were evident in the present study, the relationship between cognitive performance and total cholesterol (TC) demonstrated an intriguing pattern. Contrary to the traditional belief that hypercholesterolemia is universally harmful to brain health, the univariate regression analysis revealed a weak but significant positive association between TC levels and higher global cognitive performance (Figure 2b). Our observation of a weak positive association between total cholesterol and cognitive scores in older adults aligns with wider epidemiological syntheses, including the meta-analysis by Wee et al. (2023) [4] encompassing over 1.2 million individuals and the longitudinal findings of Anstey et al. (2017) [23] in 23,338 participants. These landmark studies demonstrate a non-linear, age-dependent shift in which midlife hypercholesterolemia increases dementia risk, whereas in late life, higher cholesterol levels may reflect nutritional adequacy, somatic robustness, or prodromal reverse causation.

The present study reflects this pattern. In the analyzed cohort, 32 participants (23.4%) with cardiovascular disease (CVD) exhibited significantly lower total cholesterol concentrations than those without CVD (177.39 vs. 201.58 mg/dL, *p* < 0.05). However, despite having lower cholesterol levels, these participants demonstrated significantly poorer cognitive performance (mean ACE-III score of 81.47 vs. 87.62, *p* = 0.003). While this apparent paradox could theoretically be linked to the frequent use of lipid-lowering therapies in the high-risk group (35.8% of the cohort, *n* = 49, were actively treated with statins), this explanation remains highly speculative. Furthermore, detailed pharmacological information regarding statin therapy—such as pre-treatment lipid baselines, treatment adherence, specific statin classification (lipophilic vs. hydrophilic), exact dosage, and duration of treatment—was not available. Consequently, a direct causal mechanism between statin therapy and the observed cholesterol-cognition paradox in the CVD subgroup cannot be definitively established. Moreover, large-scale randomized controlled trials and systematic reviews do not support an average adverse cognitive effect of statins [5,11]. Therefore, statin therapy should be considered merely as one of several potential confounding factors or co-existing hypotheses. Alternative explanations, including late-life prodromal decline in cholesterol levels, cumulative vascular burden, diabetes, frailty, or confounding by indication, must be considered highly plausible for this cross-sectional observation.

Beyond the potential confounding effect of lipid-lowering therapy, several alternative epidemiological and biological mechanisms must be considered to explain the positive association between total cholesterol and cognitive performance in this late-life cohort.

First, reverse causation represents a highly plausible explanation. Early-stage, prodromal neurodegenerative processes often trigger subtle behavioral and metabolic changes, including reduced dietary intake, altered appetite, and unintentional weight loss, which may induce a systematic decline in serum lipid levels years before clinical cognitive impairment becomes fully manifest. In this scenario, lower cholesterol levels do not actively drive cognitive decline but rather serve as a secondary metabolic marker of pre-existing, subclinical neurodegeneration [23].

Second, nutrition-related confounding bias may underlie the observed relationship. In older populations, higher total cholesterol is frequently a surrogate marker for superior nutritional status, better self-care, and robust general health. Conversely, individuals experiencing early cognitive deficits may exhibit compromised dietary habits, leading to subclinical malnutrition, frailty, and a subsequent drop in total cholesterol. Thus, the positive correlation between cholesterol and ACE-III scores may be mediated by overall nutritional well-being rather than any direct neuroprotective properties of circulating lipids [11].

Finally, the influence of survivor bias (selective survival) must also be considered. Midlife hypercholesterolemia is an established risk factor for cardiovascular disease and premature mortality [4]. Individuals highly susceptible to the deleterious vascular effects of elevated lipids may have died prematurely or developed severe cardiovascular complications that disqualified them according to our strict exclusion criteria (such as unstable coronary artery disease or advanced heart failure). Consequently, participants who reached late middle and older age with elevated cholesterol levels without manifesting major organ damage may represent a select, biologically resilient subpopulation of “healthy survivors.” In these individuals, preserved cognitive function coexists with higher lipid levels, creating an apparent yet non-causal positive association [5].

Several limitations of the study should be considered when interpreting the findings.

First, given the exploratory, cross-sectional design of this study, establishing direct cause-and-effect relationships or determining whether metabolic parameters predict future cognitive decline is precluded. Importantly, the absence of multivariable adjustment represents a major limitation. Because our analysis reports unadjusted cross-sectional associations, whether fasting glucose and total cholesterol represent independent predictors of cognitive performance after controlling for major confounding variables—such as age, formal education level, diabetes status, smoking status, lipid-lowering therapy, or depressive symptoms—cannot be determined.

Second, the evaluation of cardiometabolic risk factors relied on single fasting blood measurements of glucose and total cholesterol. Continuous glucose monitoring or assessments of long-term lipid trajectories were not employed, which would better capture acute glycemic variability, unrecognized hypoglycemic episodes, or historical metabolic fluctuations known to significantly affect brain health. Furthermore, the potential confounding effects of polypharmacy were not fully controlled for. Although the vast majority of participants were regularly taking medications, including antidiabetic agents and statins, detailed data regarding specific drug types (e.g., hydrophilic vs. lipophilic statins), dosages, and treatment duration were unavailable. This limits our ability to evaluate potential medication-induced cognitive adverse effects, such as those highlighted by the 2012 FDA warning regarding statin-associated reversible memory loss [24,25]. Additionally, cognitive performance was assessed using screening instruments (the MMSE and ACE-III) rather than comprehensive neuropsychological test batteries, and the absence of neuroimaging precludes correlating these screening scores with structural brain abnormalities.

Third, due to the exploratory nature of this investigation, no formal sample size or power calculations were performed prior to the study. The sample size of *n* = 137 was determined solely by the recruitment capacity of a single primary care facility within a specified timeframe. Although a retrospective post hoc power analysis confirmed approximately 90% statistical power for the primary glucose–ACE-III association (R^2^ = 0.069), the study may be underpowered to detect weaker effect sizes or secondary subgroup interactions. Consequently, secondary analyses may have possessed limited statistical power to detect smaller effect sizes. This issue is further compounded by multiple statistical comparisons performed across various metabolic, vascular, and psychosocial parameters without formal multiplicity corrections, thereby increasing the overall risk of type I errors. Therefore, our findings must be interpreted cautiously as exploratory and hypothesis-generating.

Fourth, potential selection and generalizability biases must be acknowledged. Recruitment was volunteer-based among patients attending consecutive family medicine clinics, thereby potentially introducing selection bias. Individuals experiencing subjective memory concerns or those with greater leisure time, specifically retired or professionally inactive individuals, who constituted 72.3% of our cohort, may have been more motivated to participate. Additionally, because participants were consecutively recruited from a single primary care clinic, without a healthy control group, and presented with a high burden of chronic comorbidities, the generalizability of our findings to broader, healthier, or younger populations is limited. However, this highly selected, multimorbid cohort accurately reflects the real-world clinical population managed daily in primary care practice. To confirm these preliminary findings, larger prospective longitudinal studies incorporating standardized long-term metabolic monitoring and comprehensive neuropsychological assessments are warranted.

Additionally, our metabolic characterization was limited by the reliance on fasting glucose without glycated hemoglobin (HbA1c) data, precluding the evaluation of chronic glycemic exposure. Similarly, total cholesterol values frequently reflect treated states, as historical pre-treatment lipid levels, statin adherence, and specific dosage information were unavailable. Finally, although lifestyle and psychosocial parameters (such as physical activity, computer use, and depressive symptoms) were recorded in our wider research project, they were not statistically adjusted for in the present regression models and must be interpreted strictly as non-causal, historical associations.

## 5. Conclusions

Elevated fasting blood glucose (FBG) levels were associated with poorer cognitive performance in this primary care cohort. Routine evaluation of fundamental metabolic parameters, such as FBG, which represent simple and widely available clinical measures, may assist in identifying individuals with concurrent cognitive impairment in primary care settings, although longitudinal studies are required to establish their predictive value.

The relationship between total cholesterol and cognition in older adults is complex. In this study, lower total cholesterol levels were associated with worse cognitive performance. This apparent clinical paradox cannot be definitively attributed to lipid-lowering therapies (statins), as our cross-sectional data lack detailed treatment parameters and randomized controlled trial evidence does not support a causal adverse effect. Instead, statin use—along with late-life prodromal lipid decline, cumulative vascular burden, and age-related comorbidities—should be regarded as potential confounding factors or hypotheses warranting further prospective longitudinal investigation. The potential clinical value of routine metabolic markers for preliminary cognitive risk stratification in primary care remains strictly at the hypothesis-generating stage. Rigorous prospective longitudinal validation with repeated metabolic and cognitive tracking is required before these routine parameters can serve as formal clinical screening indicators. Further longitudinal studies incorporating long-term metabolic monitoring are required to establish causality.

Given the observational cross-sectional design, no causal inferences can be drawn regarding the protective or deleterious effects of these metabolic parameters, and all findings remain strictly exploratory due to the absence of multiplicity adjustments. Therefore, prospective longitudinal studies incorporating long-term metabolic monitoring are essential to establish causality.

Consistent with previously reported associations in this cohort, engagement in regular physical activity and frequent computer use—alongside fewer depressive symptoms—were associated with better cognitive performance, although our cross-sectional design precludes establishing a causal protective effect.

Importantly, standard screening tools such as the MMSE exhibited disparate patterns of association compared with more comprehensive instruments like the ACE-III in this setting. In line with the 2026 Clinical Guidelines of Diabetes Poland (PTD) [19], integrating cognitive screening with routine metabolic monitoring in the primary care population remains a promising research direction, although any clinical utility for the early diagnosis or targeted prevention of neurocognitive disorders (NCDs) warrants formal prospective validation.

## Figures and Tables

**Figure 1:**
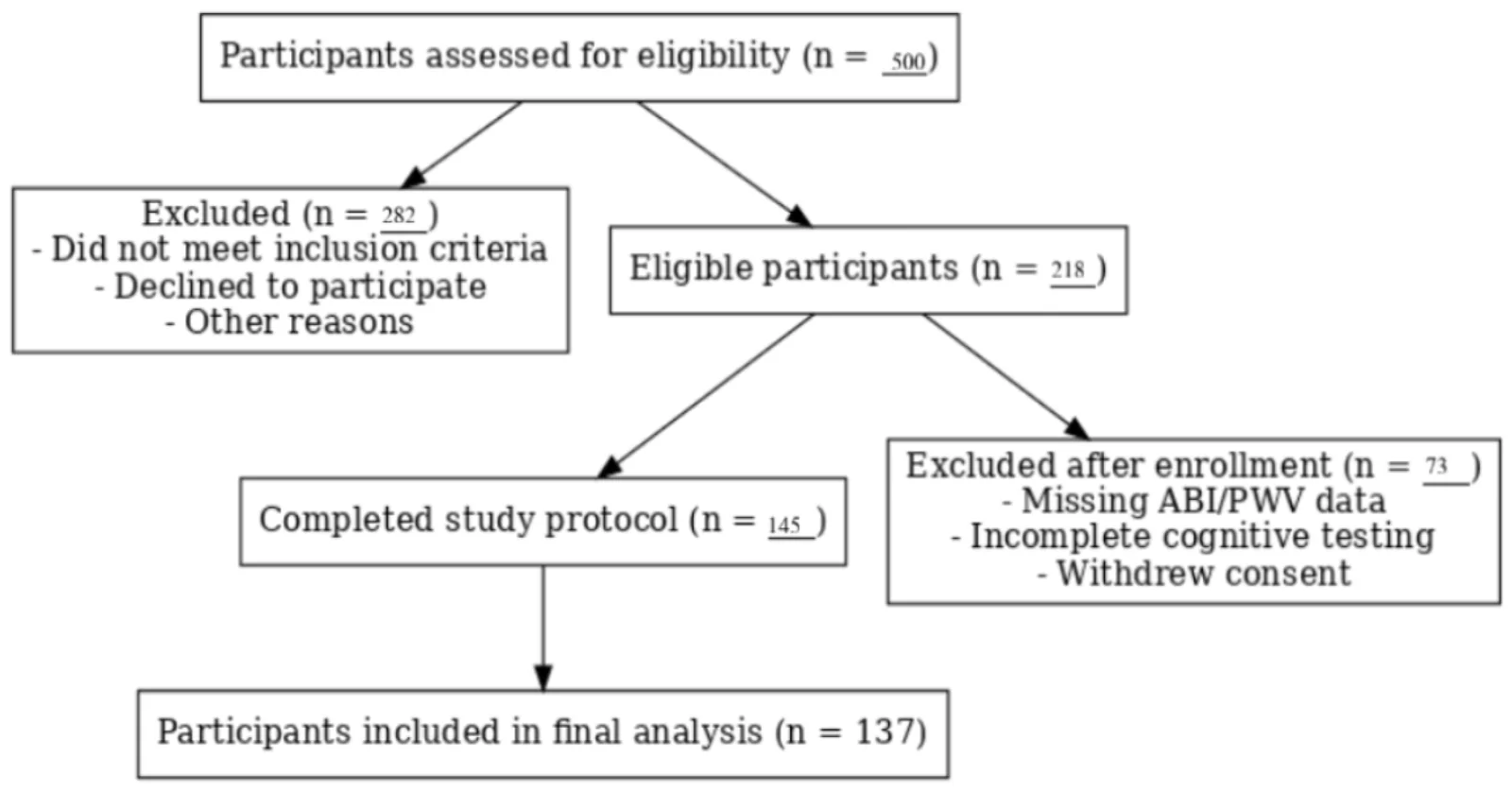
A flowchart of participant recruitment, exclusion, and inclusion. The figure illustrates the number of individuals assessed for eligibility, reasons for exclusion, and the final number of participants included in the analysis (*n* = 137).

**Figure 2:**
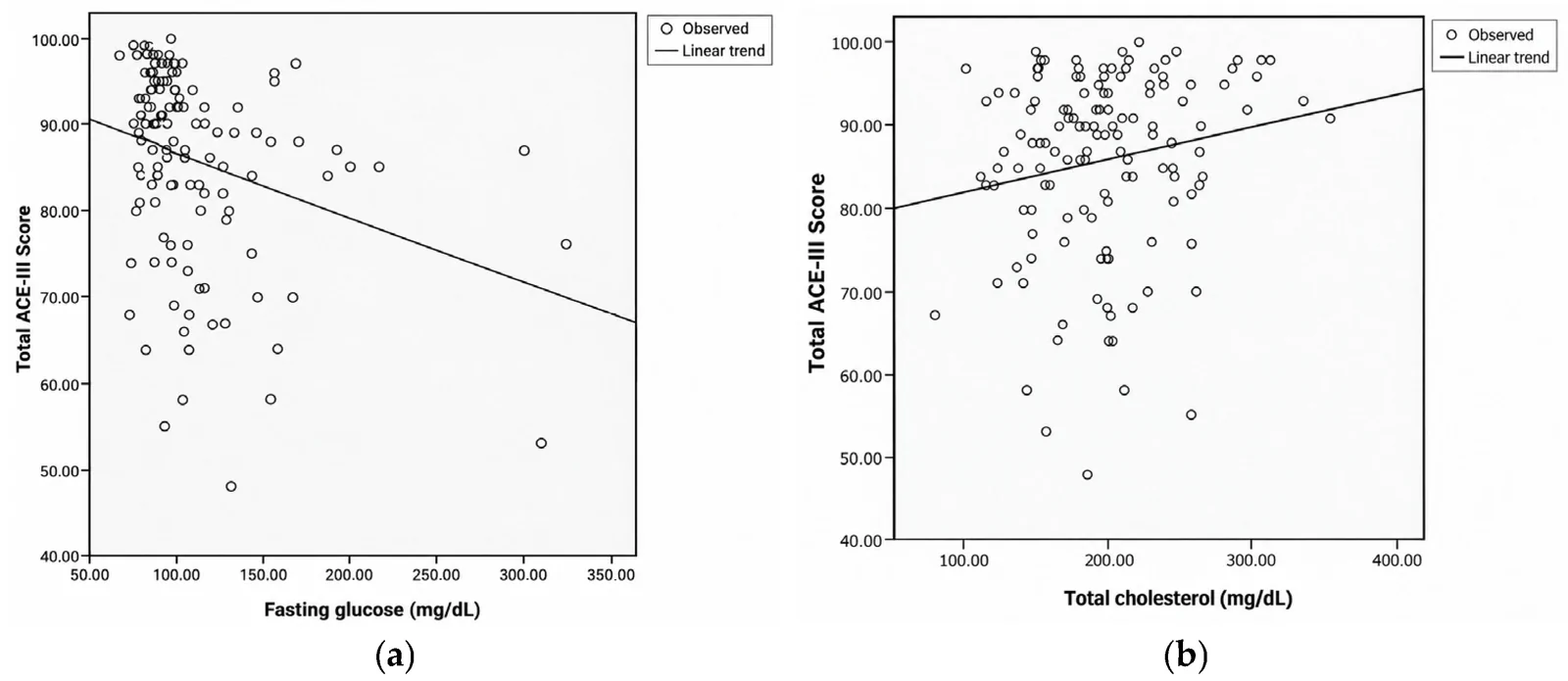
(**a**) The relationship between the dependent variable ACE-III score (0–100 points) and the independent variable fasting glucose (mg/dL), demonstrating a weak but significant negative association (standardized β = −0.277, 95% CI: −0.44 to −0.11, unstandardized B = −0.075, *p* = 0.001; adjusted R^2^ = 0.069, F(1,129) = 10.704, *p* < 0.05). The regression line is plotted with 95% confidence bands. (**b**) The relationship between the dependent variable ACE-III score (0–100 points) and the independent variable total cholesterol (mg/dL), demonstrating a weak but significant positive association (standardized β = 0.173, 95% CI: 0.01 to 0.34, unstandardized B = 0.039, *p* = 0.046; adjusted R^2^ = 0.022, F(1,132) = 4.059, *p* < 0.05). The regression line is plotted with 95% confidence bands.

**Figure 3:**
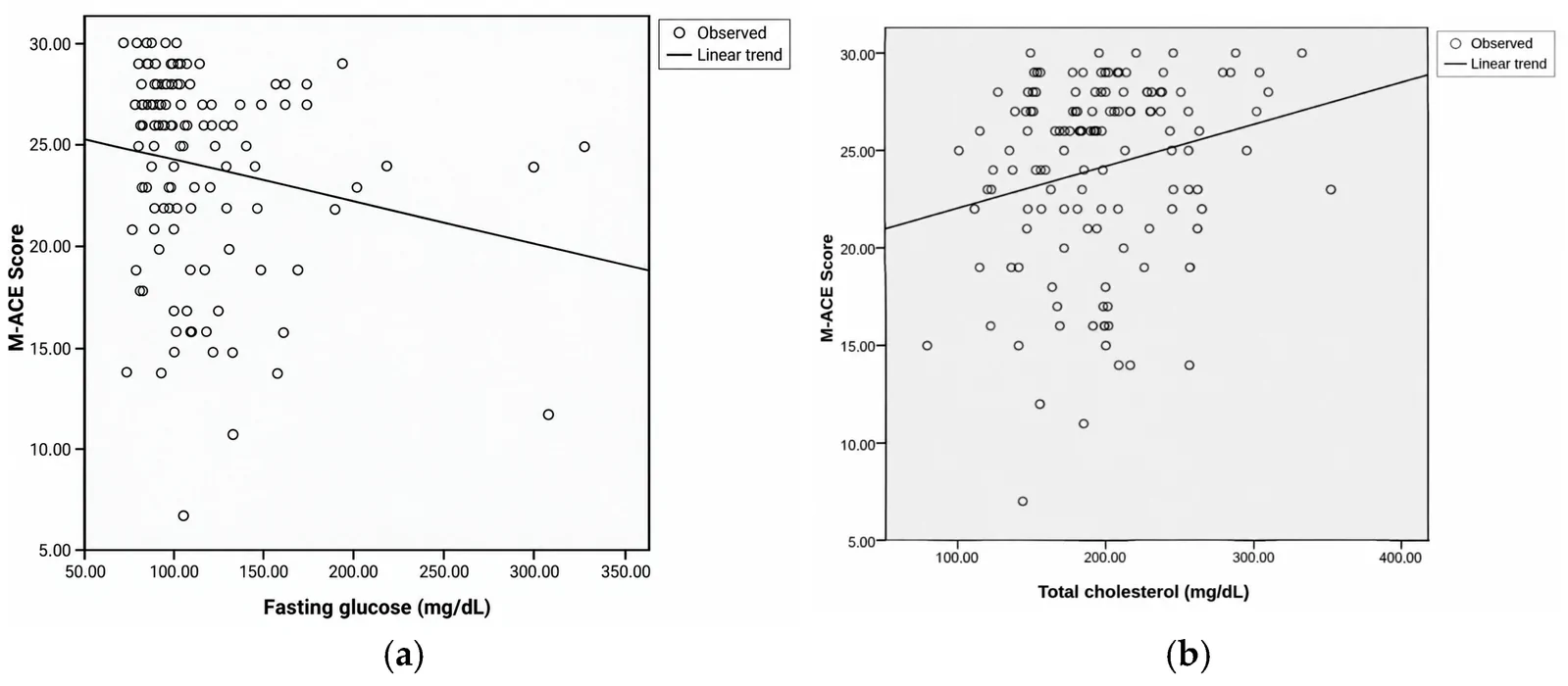
(**a**) The relationship between the dependent variable M-ACE score and the independent variable fasting glucose (mg/dL), demonstrating a weak but significant negative association (standardized β = −0.175, 95% CI: −0.35 to −0.01, unstandardized B = −0.020, *p* = 0.046; adjusted R^2^ = 0.023, F(1,129) = 4.052, *p* < 0.05). The regression line is plotted with 95% confidence bands. (**b**) The relationship between the dependent variable M-ACE score (0–30 points) and the independent variable total cholesterol (mg/dL), demonstrating a weak but significant positive association (standardized β = 0.219, 95% CI: 0.05 to 0.39, unstandardized B = 0.022, *p* = 0.011; adjusted R^2^ = 0.041, F(1,132) = 6.673, *p* < 0.05). The regression line is plotted with 95% confidence bands.

**Table 1:** Baseline demographic, metabolic, and cognitive characteristics of the study population (*n* = 137).

Variable	Unit/Metric	Mean (SD)/Percentage
*Demographics and Anthropometrics*		
Age	years, mean (SD)	63.46 (5.53)
Females	*n* (%)	73 (53.3%)
Males	*n* (%)	64 (46.7%)
BMI	(kg/m^2^), mean (SD)	27.77 (5.49)
*Metabolic profile*		
Fasting glucose	(mg/dL), mean (SD)	113.02 (41.11)
Total cholesterol	(mg/dL), mean (SD)	195.99 (49.04)
LDL cholesterol	(mg/dL), mean (SD)	111.71 (40.17)
HDL cholesterol	(mg/dL), mean (SD)	56.37 (23.29)
Triglycerides	(mg/dL), mean (SD)	147.41 (95.02)
*Cognitive Functioning*		
MMSE total score (0–30 points)	points (0–30), mean (SD)	28.07 (5.11)
ACE-III total score (0–100 points)	points (0–100), mean (SD)	86.04 (11.04)
M-ACE (0–30 points)	points (0–30), mean (SD)	24.20 (4.79)

**Table 2:** Psychosocial and socioeconomic profile of the study population (*n* = 137).

Variable	Quantity (*n*)	Percentage (%)
*Social Support & Environment*		
Living alone	27	19.71
Recent loss of a close friend	27	19.71
No one to confide in	20	14.60
Major marital conflicts	7	5.11
Able to share emotions openly	103	75.18
*Emotional Well-Being*		
History of traumatic events	57	41.61
Feelings of hopelessness	35	25.55
Loss of pleasure in daily life	18	13.14
Symptoms of anxiety	23	16.79
Excessive worrying	26	18.98
Frequent anger over trivial matters	39	28.47
Easily annoyed by others	48	35.04
*Socioeconomic & Occupational Factors*		
Satisfied with current income	79	57.66
Inadequate material situation	24	17.52
Occupational stress (loss of control)	19	13.87
*Lifestyle Choices*		
Physical activity (min. once a week)	65	47.45
Regular computer use	85	62.04
*Clinical History*		
Past or current psychiatric disorder	30	21.90
Central nervous system diseases	8	5.84
Family history of dementia	14	10.22
*Medication*		
Sedative and hypnotic medications	36	26.28
Pro-cognitive agents	22	16.06
Antidepressants	19	13.87
Neuroleptics	15	10.95
Anticonvulsant medications	8	5.84

Note: The 22 participants (16.06%) using pro-cognitive agents predominantly utilized over-the-counter or prescribed Ginkgo biloba extracts, piracetam, or vinpocetine for subjective complaints (e.g., tinnitus, subjective memory decline) without any formal, pre-existing objective diagnosis of cognitive impairment.

## Data Availability

The individual-level, fully de-identified dataset supporting the findings of this study (combining cognitive screening scores, laboratory metrics, and vascular values) is openly available in Zenodo at https://doi.org/10.5281/zenodo.14919033. To ensure participant anonymity and prevent any risk of re-identification in strict compliance with GDPR regulations, all direct and indirect personal identifiers (including exact dates of birth, clinical visit dates, and specific geographic details) were systematically removed prior to deposition. Unrestricted public release of these anonymized research data was covered by the participants’ original informed consent and formally approved by the Bioethics Committee of the Poznan University of Medical Sciences under resolution number 1061/16.

## Abbreviations

The following abbreviations are used in this manuscript:

ADOD	Alzheimer’s Disease and Other Dementias
MCI	Mild Cognitive Impairment
MMSE	Mini-Mental State Examination
ACE-III	Addenbrooke’s Cognitive Examination III
NCD	Neurocognitive Disorder
CVD	Cardiovascular Disease
ABI	Ankle–Brachial Index
PWV	Pulse Wave Velocity
TC	Total Cholesterol
LDL	Low-Density Lipoprotein
VLDL	Very-Low-Density Lipoprotein
HDL	High-Density Lipoprotein
FBG	Fasting Blood Glucose
BMI	Body Mass Index
ESH	European Society of Hypertension
PTD	Polskie Towarzystwo Diabetologiczne/Polish Society of Diabetes
